# Designing and evaluating the MULTICOM protein local and global model quality prediction methods in the CASP10 experiment

**DOI:** 10.1186/1472-6807-14-13

**Published:** 2014-04-15

**Authors:** Renzhi Cao, Zheng Wang, Jianlin Cheng

**Affiliations:** 1 Computer Science Department, University of Missouri, Columbia, Missouri 65211, USA; 2 Informatics Institute, University of Missouri, Columbia, Missouri 65211, USA; 3 Christopher S. Bond Life Science Center, University of Missouri, Columbia, Missouri 65211, USA; 4 School of Computing, University of Southern Mississippi, Hattiesburg, MS 39406-0001, USA

**Keywords:** Protein model quality assessment, Protein model quality assurance program, Protein structure prediction, Support vector machine, Clustering

## Abstract

**Background:**

Protein model quality assessment is an essential component of generating and using protein structural models. During the Tenth Critical Assessment of Techniques for Protein Structure Prediction (CASP10), we developed and tested four automated methods (MULTICOM-REFINE, MULTICOM-CLUSTER, MULTICOM-NOVEL, and MULTICOM-CONSTRUCT) that predicted both local and global quality of protein structural models.

**Results:**

MULTICOM-REFINE was a clustering approach that used the average pairwise structural similarity between models to measure the global quality and the average Euclidean distance between a model and several top ranked models to measure the local quality. MULTICOM-CLUSTER and MULTICOM-NOVEL were two new support vector machine-based methods of predicting both the local and global quality of a single protein model. MULTICOM-CONSTRUCT was a new weighted pairwise model comparison (clustering) method that used the weighted average similarity between models in a pool to measure the global model quality. Our experiments showed that the pairwise model assessment methods worked better when a large portion of models in the pool were of good quality, whereas single-model quality assessment methods performed better on some hard targets when only a small portion of models in the pool were of reasonable quality.

**Conclusions:**

Since digging out a few good models from a large pool of low-quality models is a major challenge in protein structure prediction, single model quality assessment methods appear to be poised to make important contributions to protein structure modeling. The other interesting finding was that single-model quality assessment scores could be used to weight the models by the consensus pairwise model comparison method to improve its accuracy.

## Background

Predicting protein tertiary structure from amino acid sequence is of great importance in bioinformatics and computational biology [1,2]. During the last few decades, a lot of protein tertiary structure prediction methods have been developed. One category of methods adopts a template-based approach [3-7], which uses experimentally determined structures as templates to build structural models for a target protein without known structure. Another category uses a template-free approach [8,9], which tries to fold a protein from scratch without using known template structures. The two kinds of methods were often combined to handle a full spectrum of protein structure prediction problems ranging from relatively easy homology modeling to hard *de novo* prediction [10-13].

During protein structure prediction, one important task is to assess the quality of structural models produced by protein structure prediction methods. A model quality assessment (QA) method employed in a protein structure prediction pipeline is critical for ranking, refining, and selecting models [3]. A model quality assessment method can generally predict a global quality score measuring the overall quality of a protein structure model and a series of local quality scores measuring the local quality of each residue in the model. A global quality score can be a global distance test (GDT-TS) score [14-16] that is predicted to be the structural similarity between a model and the unknown native structure of a protein. A local quality score of a residue can be the Euclidean distance between the position of the residue in a model and that in the unknown native structure after they are superimposed.

In general, protein model quality assessment methods can be classified into two categories: multi-model methods [17-21] and single-model methods [13-17]. Multi-model methods largely use a consensus or clustering approach to compare one model with other models in a pool of input models to assess its quality. Generally, a model with a higher similarity with the rest of models in the pool receives a higher global quality score. The methods tend to work well when a large portion of models in the input pool are of good quality, which is often the case for easy to medium hard template-based modeling. Multi-model methods tend to work particularly well if a large portion of good models were independently generated by a number of independent, diverse protein structure prediction methods as seen in the CASP (the Critical Assessment of Techniques for Protein Structure Prediction) experiments, but they worked less well when being applied to the models generated by one single protein structure prediction method because they prefer the average model of the largest model cluster in the model pool. And multi-model methods tend to completely fail if a significant portion of low quality modes are similar to each other and thus dominate the pairwise model comparison as seen in some cases during the 10^th^ CASP experiment (CASP10) held in 2012. Single-model methods strive to predict the quality of a single protein model without consulting any other models [22-26]. The performance of single-model methods is still lagging behind the multi-model methods in most cases when most models in the pool are of good quality [23,27]. However, because of their capability of assessing the quality of one individual model, they have potential to address one big challenge in protein structure modeling – selecting a model of good quality from a large pool consisting of mostly irrelevant models. Furthermore, as the performance of multi-model quality assessment methods start to converge, single-model methods appear to have a large room of improvement as demonstrated in the CASP10 experiment.

In order to critically evaluate the performance of multi-model and single-model protein model quality assessment methods, the CASP10 experiment was designed to assess them in two stages. On Stage 1, 20 models of each target spanning a wide range of quality were used to assess the sensitivity of quality assessment methods with respect to the size of input model pool and the quality of input models. On Stage 2, about top 150 models selected by a naïve consensus model quality assessment method were used to benchmark model quality assessment methods’ capability of distinguishing relatively small differences between more similar models. The new settings provided us a good opportunity to assess the strength and weakness of our multi-model and single-model protein model quality assessment methods in terms of accuracy, robustness, consistency and efficiency in order to identify the gaps for further improvement.

In addition to evaluating our four servers on the CASP10 benchmark, we compare our methods with three popular multi-model clustering-based methods (Davis-QAconsensus [28], Pcons [29], and ModFOLDclust2 [21]). Our clustering-based methods (MULTICOM-REFINE, MULTICOM-CONSTRUCT) performed comparably to the three external tools in most cases. Our single-model methods (MULTICOM-CLUSTER, MULTICOM-NOVEL) had a lower accuracy than the clustering-based methods, but performed considerably better than them on the models of hard template-free targets. Besides the reasonable performance and a comprehensive comparative study, our methods have some methodological innovations such as using single-model quality scores to weight models for clustering methods, repacking side chains before model evaluation, and improved machine learning methods for single-model quality assessment for template-free targets.

The rest of the paper is organized as follows. In the Results and discussions section, we analyze and discuss the performance of the methods on the CASP10 benchmark. In the Conclusion section, we summarize this work and conclude it with the directions of future work. In the Methods section, we introduce the methods in our protein model quality assessment servers tested in CASP10.

## Results and discussions

### Results of global quality predictions

We evaluated the global quality predictions using five measures (see the detailed descriptions of the evaluation methods in the Evaluation methods section). The results of the global quality evaluation on Stage 1 of CASP10 are shown in Table 1. The weighted pairwise model comparison method MULTICOM-CONSTRUCT performed best among all our four servers according to all the five measures, suggesting using single-model quality prediction scores as weights can improve the multi-model pairwise comparison based quality prediction methods such as MULTICOM-REFINE. The two multi-model global quality assessment methods had the better average performance than the two single-model global quality assessment methods (MULTICOM-NOVEL and MULTICOM-CLUSTER) on average on Stage 1, suggesting that the advantage of multi-model methods over single-model methods was not much affected by the relatively small size of input models (i.e. 20). Instead, the multi-model methods still work reasonably well on a small model pool that contains a significant portion of good quality models. It is worth noting that the average loss of the two single-model quality assessment methods (MULTICOM-CLUSTER and MULTICOM-NOVEL) is close to that of the two multi-model quality assessment methods (MULTICOM-REFINE and MULTICOM-CONSTRUCT) (i.e. +0.07 versus +0.06). We also compared our methods with three popular multi-model clustering-based methods (DAVIS-QAconsensus, Pcons, and ModFOLDclust2) on Stage 1. According to the evaluation, MULTICOM-CONSTRUCT performed slightly better than the naive consensus method DAVIS-QAconsensus and ModFOLDclust2, while Pcons performed best.

**Table 1:** **The average correlation (Ave. Corr.), overall correlation** (**Over. Corr**.)**, average GDT-TS loss (Ave. Loss**), **average Spearman**’**s correlation** (**Ave. Spearman**), **average Kendall tau correlation** (**Ave. Kendall**) **of MULTICOM servers**, **DAVIS**-**QAconsensus**, **Pcons**, **and ModFOLDclust2 on Stage 1 of CASP10**

**Servers**	**Ave. corr.**	**Over. corr.**	**Ave. loss**	**Ave. Spearman**	**Ave. Kendall**
MULTICOM-REFINE	0.6494	0.8162	0.0615	0.5989	0.4908
MULTICOM-CLUSTER	0.5144	0.5946	0.0727	0.4364	0.3273
MULTICOM-NOVEL	0.5016	0.4848	0.0791	0.4483	0.3380
MULTICOM-CONSTRUCT	0.6838	0.8300	0.0613	0.6182	0.5043
DAVIS-QAconsensus	0.6403	0.7927	0.0537	0.5798	0.4745
Pcons	0.7501	0.7683	0.0327	0.6781	0.5457
ModFOLDclust2	0.6775	0.8301	0.0572	0.6206	0.5064

Table 2 shows the global quality evaluation results on Stage 2. Similarly as in Table 1, the weighted pairwise comparison multi-model method (MULTICOM-CONSTRUCT) performed better than the simple pairwise multi-model method (MULTICOM-REFINE) and both had better performance than the two single-model quality assessment methods (MULTICOM-CONSTRUCT and MULTICOM-NOVEL). That the two single-model quality prediction methods yielded the similar performance indicated that some difference in their input features (amino acid sequence versus sequence profile) did not significant affect their accuracy. In comparison with Stage 1, all our methods performed worse on Stage 2 models. Since the models in Stage 2 are more similar to each other than in Stage 1 in most cases, the results may suggest that both multi-model and single-model quality assessment methods face difficulty in accurately distinguishing models of similar quality. On Stage 2 models, MULTICOM-CONSTRUCT delivered a performance similar with DAVIS-QAconsensus and Pcons, and had a higher average correlation than ModFOLDclust2.

**Table 2:** **The average correlation**, **overall correlation**, **average GDT**-**TS loss**, **average Spearman**’**s correlation**, **average Kendall tau correlation of MULTICOM servers**, **DAVIS**-**QAconsensus**, **Pcons**, **and ModFOLDclust2 on Stage 2 of CASP10**

**Servers**	**Ave. corr.**	**Over. corr.**	**Ave. loss**	**Ave. Spearman**	**Ave. Kendall**
MULTICOM-REFINE	0.4743	0.8252	0.0511	0.4763	0.3510
MULTICOM-CLUSTER	0.3354	0.6078	0.0675	0.3361	0.2343
MULTICOM-NOVEL	0.3350	0.5057	0.0654	0.3394	0.2358
MULTICOM-CONSTRUCT	0.4853	0.8272	0.0510	0.4824	0.3566
DAVIS-QAconsensus	0.5050	0.8383	0.0499	0.5031	0.3686
Pcons	0.4891	0.8194	0.0416	0.4843	0.3524
ModFOLDclust2	0.4489	0.8337	0.0470	0.4621	0.3393

We used the Wilcoxon signed ranked sum test to assess the significance of the difference in the performance of our four servers, DAVIS-QAconsensus, Pcons, and ModFOLDclust2. The p-values of the difference between these servers are reported in Table 3. On Stage 1 models, according to 0.01 significant threshold, the difference between clustering-based methods (MULTICOM-REFINE and MULTICOM-CONSTRUCT) and single-model methods (MULTICOM-CLUSTER and MULTICOM-NOVEL) is significant, but the difference between our methods in the same category is not significant. One Stage 2 models, the difference between all pairs of our servers except the two single-model methods is significant. Compared with the three external methods (DAVIS-QAconsensus, Pcons, and ModFOLDclust2), the difference between our multi-model method MULTICOM-REFINE and the three methods is not significant, while the difference between our single-model methods (MULTICOM-CLUSTER, MULTICOM-NOVEL) and the three methods is significant. The difference between MULTICOM-CONSTRUCT and Pcons is not significant, while the difference between MULTICOM-CONSTRUCT and the other two external methods (DAVIS-QAconsensus and ModFOLDclust2) is significant.

**Table 3:** **The p**-**value of pairwise Wilcoxon signed ranked sum test for the difference of correlation score between MULTICOM servers and three external methods** (**DAVIS**-**QAconsensus**, **Pcons**, **ModFOLDclust2**) **on Stage 1 and Stage 2 of CASP10**

**MULTICOM servers, DAVIS-QAconsensus, Pcons, and ModFOLDclust2 on Stage 1 or Stage 2**	**P-value**
MULTICOM-REFINE and MULTICOM-CLUSTER on Stage 1	7.552e-05
MULTICOM-REFINE and MULTICOM-NOVEL on Stage 1	3.280e-05
MULTICOM-REFINE and MULTICOM-CONSTRUCT on Stage 1	0.031
MULTICOM-CLUSTER and MULTICOM-NOVEL on Stage 1	0.201
MULTICOM-CLUSTER and MULTICOM-CONSTRUCT on Stage 1	3.757e-06
MULTICOM-NOVEL and MULTICOM-CONSTRUCT on Stage 1	7.013e-07
MULTICOM-REFINE and Pcons on Stage 1	0.1723
MULTICOM-REFINE and ModFOLDclust2 on Stage 1	0.578
MULTICOM-REFINE and DAVIS-QAconsensus on Stage 1	0.6238
MULTICOM-CLUSTER and Pcons on Stage 1	2.872e-08
MULTICOM-CLUSTER and ModFOLDclust2 on Stage 1	5.517e-05
MULTICOM-CLUSTER and DAVIS-QAconsensus on Stage 1	0.002873
MULTICOM-NOVEL and Pcons on Stage 1	5.65e-09
MULTICOM-NOVEL and ModFOLDclust2 on Stage 1	2.116e-05
MULTICOM-NOVEL and DAVIS-QAconsensus on Stage 1	0.002066
MULTICOM-CONSTRUCT and Pcons on Stage 1	0.7492
MULTICOM-CONSTRUCT and ModFOLDclust2 on Stage 1	0.01223
MULTICOM-CONSTRUCT and DAVIS-QAconsensus on Stage 1	0.0002211
MULTICOM-REFINE and MULTICOM-CLUSTER on Stage 2	4.133e-05
MULTICOM-REFINE and MULTICOM-NOVEL on Stage 2	3.180e-05
MULTICOM-REFINE and MULTICOM-CONSTRUCT on Stage 2	2.439e-05
MULTICOM-CLUSTER and MULTICOM-NOVEL on Stage 2	0.658
MULTICOM-CLUSTER and MULTICOM-CONSTRUCT on Stage 2	7.75e-06
MULTICOM-NOVEL and MULTICOM-CONSTRUCT on Stage 2	5.276e-06
MULTICOM-REFINE and Pcons on Stage 2	0.2465
MULTICOM-REFINE and ModFOLDclust2 on Stage 2	0.08742
MULTICOM-REFINE and DAVIS-QAconsensus on Stage 2	0.4976
MULTICOM-CLUSTER and Pcons on Stage 2	1.114e-05
MULTICOM-CLUSTER and ModFOLDclust2 on Stage 2	0.001202
MULTICOM-CLUSTER and DAVIS-QAconsensus on Stage 2	7.495e-06
MULTICOM-NOVEL and Pcons on Stage 2	1.073e-05
MULTICOM-NOVEL and ModFOLDclust2 on Stage 2	0.001128
MULTICOM-NOVEL and DAVIS-QAconsensus on Stage 2	5.717e-06
MULTICOM-CONSTRUCT and Pcons on Stage 2	0.9807
MULTICOM-CONSTRUCT and ModFOLDclust2 on Stage 2	0.003362
MULTICOM-CONSTRUCT and DAVIS-QAconsensus on Stage 2	9.597e-05

**Figure 1:**
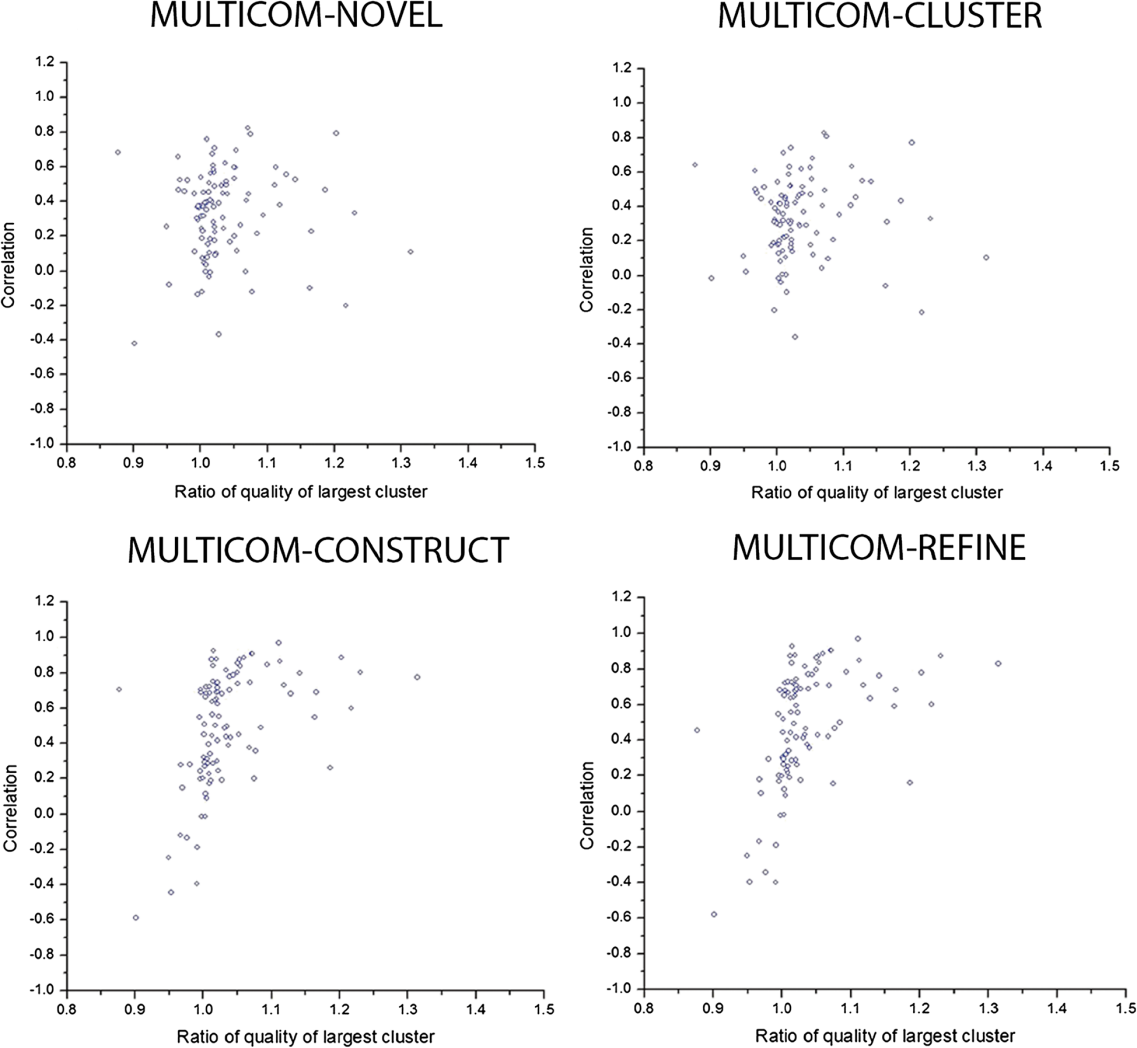
The per-target correlation scores of each target against the average real quality of the largest model cluster divided by the average real quality of all models of this target on Stage 2.

To elucidate the key factors that affect the accuracy of multi-model or single-model quality assessment methods, we plot the per-target correlation scores of each target on Stage 2 against the ratio of the average real quality of the largest model cluster in the pool and the average real quality of all the models in the pool in Figure 1. To get the largest model cluster for each target, we first calculate the GDT-TS score between each pair of models, and then use (1 – the GDT-TS score) as the distance measure to hierarchically cluster the models. Finally, we use a distance threshold to cut the hierarchical tree to get the largest cluster so that the total number of models in the largest cluster is about one third of the total number of models in the pool.

Figure 1 shows that the quality prediction accuracy (i.e. per-target correlation scores of each target) positively correlates with the average real quality of the largest model cluster divided by the average real quality of all models for two multi-model methods (MULTICOM-REFINE, MULTICOM-CONSTRUCT), whereas it has almost no correlation with single-model methods (MULTICOM-CLUSTER, MULTICOM-NOVEL). The results suggest that the performance of clustering-based multi-model methods depends on the relative real quality of the large cluster of models and that of single-model methods does not. This is not surprising because multi-model methods rely on pairwise model comparison, but single-model methods try to assess the quality from one model.

**Figure 2:**
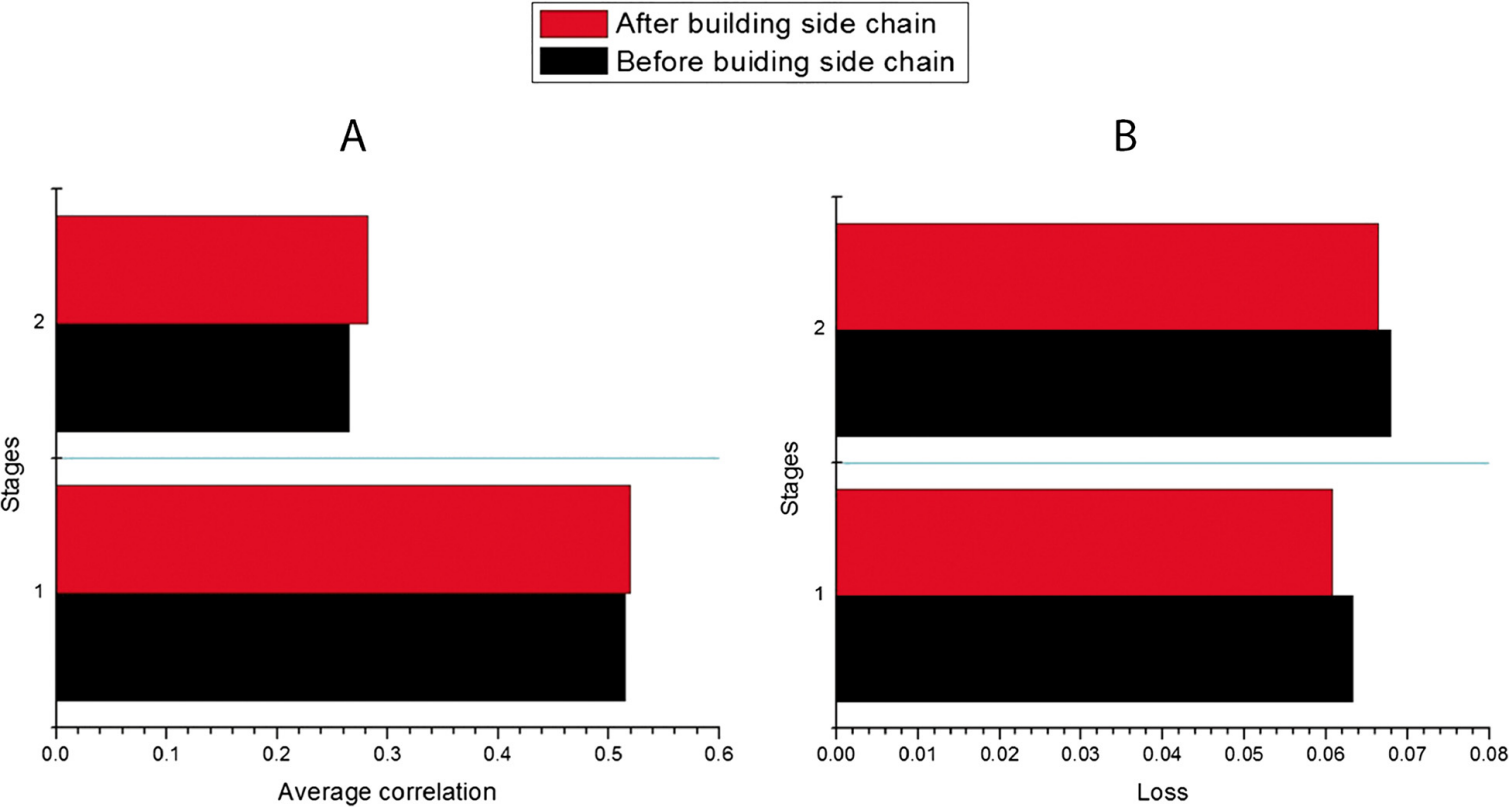
**The influence of side chain on average correlation and loss of both Stage 1 and Stage 2. A** shows the average correlation of the predictions with or without side-chain repacking, and **B** demonstrates the loss of the predictions with or without side-chain repacking on both Stage 1 and Stage 2. The tool SCWRL [30] is used for the side-chain repacking.

As CASP10 models were generated by many different predictors from around of the world, the side chains of these models may be packed by different modeling tools. The difference in side chain packing may result in difference in input features (e.g. secondary structures) that affect the quality prediction results of single-model methods even though they only try to predict the quality of backbone of a model. In order to remove the side-chain bias, we also tried to use the tool SCWRL [30] to rebuild the side chains of all models before applying a single-model quality prediction method - ModelEvaluator. Figure 2 compares the average correlation and loss of the predictions with or without side-chain repacking. Indeed, repacking side-chains before applying single-model quality assessment increased the average correlation and reduced the loss. We did a Wilcoxon signed ranked sum test on the correlations and losses of the predictions before and after repacking side-chains. The p-value for average correlation before and after repacking side-chains on Stage 1 is 0.18, and on Stage 2 is 0.02. The p-value for loss on Stage 1 is 0.42, and on Stage 2 is 0.38.

**Figure 3:**
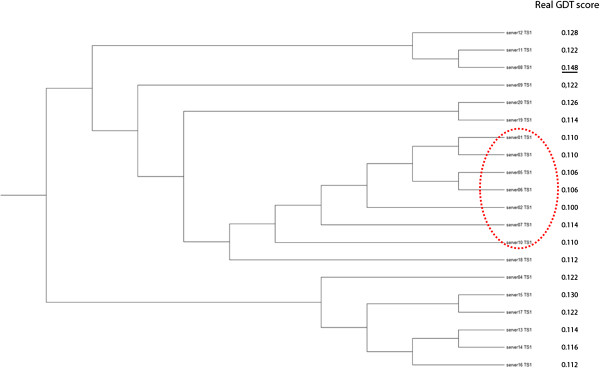
**The hierarchy tree of T0741 on Stage 1.** All models in the circle form the largest cluster in this target. The rightmost column of Figure 3 lists the real GDT-TS score of each model. The models in the circle form the largest cluster. The model with the underline real GDT-TS score is the best model in this target.

**Figure 4:**
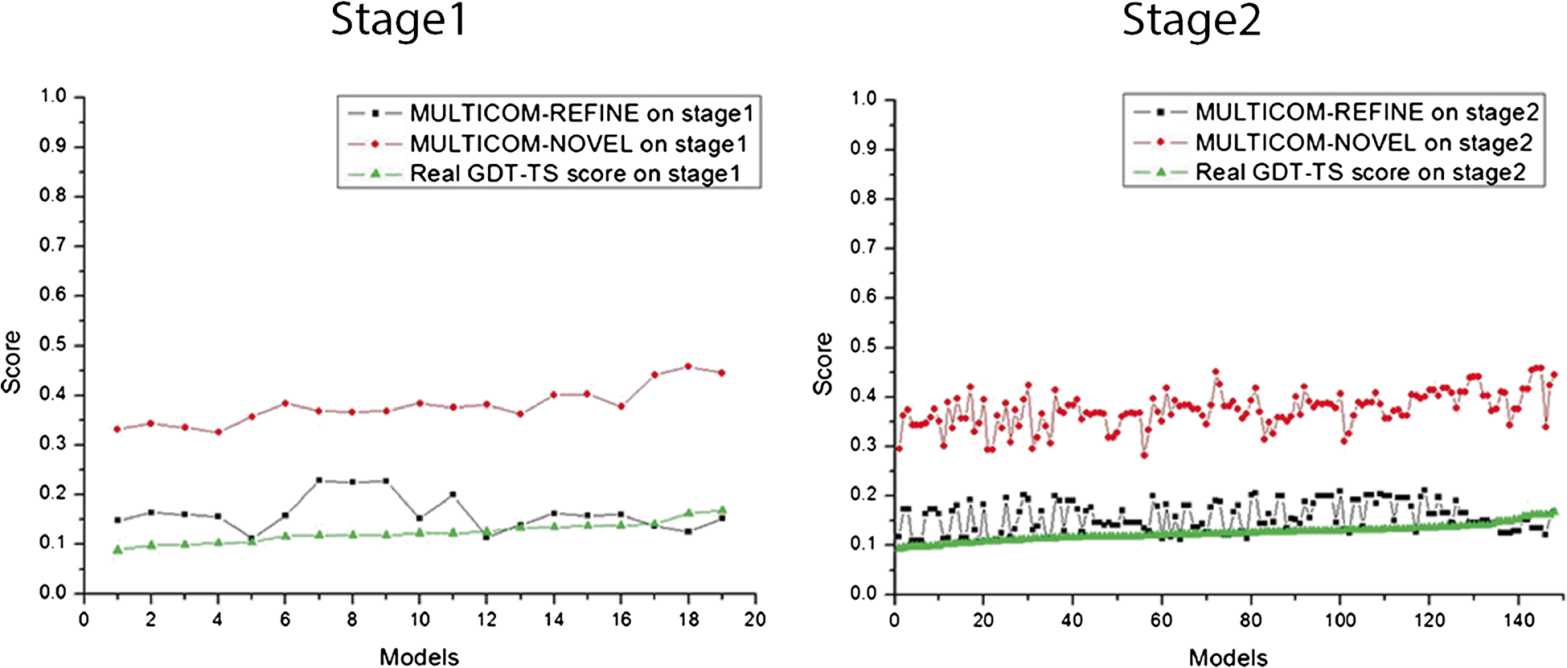
The real GDT-TS score and predicted GDT-TS score of MULTICOM-REFINE and MULTICOM-NOVEL for T0684 on Stage 1 and Stage 2.

Since mining a few good models out of a large pool of low-quality models is one of the major challenges in protein structure prediction, we compare the performance of single-model methods and multi-model methods on the models of several hard CASP10 template-free targets. Tables 4 and 5 report the evaluation results of our four servers, DAVIS-QAconsensus, Pcons, and ModFOLDclust2 on all standalone template-free modeling (FM) targets on Stages 1 and 2, i.e. the targets whose domains are all FM domains. The results show that the single-model methods (MULTICOM-CLUSTER and MULTICOM-NOVEL) clearly performed better than the multi-model methods (MULTICOM-REFINE and MULTICOM-CONSTRUCT) on both stages. They also performed better than the DAVIS-QAconsensus and ModFOLDclust2 on both stages, achieved the similar performance with Pcons on Stage 1, and the better performance than Pcons on Stage 2. For instance, the average Pearson’s correlation score of MULTICOM-NOVEL on Stage 1 is 0.539, which is much higher than 0.082 of MULTICOM-REFINE. The multi-model methods even get low negative correlation for some targets. For example, the Pearson’s correlation score of MULTICOM-REFINE on target T0741 at Stage 1 is -0.615. We use the tool TreeView [31] to visualize the hierarchical clustering of the models of T0741 in Figure 3. The qualities of the models in the largest cluster are among the lowest, but they are similar to each other leading to high predicted quality scores when being assessed by multi-model methods. The example indicates that multi-model methods often completely fail (i.e. yielding negative correlation) when the models in the largest cluster are of worse quality, but similar to each other. Multi-model methods often perform worse than single-model methods when all models in pool are of low quality and are different from each other. In this situation, the quality scores predicted by multi-model methods often do not correlate with the real quality scores, whereas those predicted by single-model methods still positively correlate with real quality scores to some degree. As an example, Figure 4 plots the real GDT-TS scores and predicted GDT-TS scores of a single-model predictor MULTICOM-NOVEL and a multi-model predictor MULTICOM-REFINE on the models of a hard target T0684 whose best model has quality score less than 0.2. It is worth noting that, since the quality of the models of the template-free modeling targets is rather low on average, the quality assessment on these models can be more arbitrary than on the template-based models of better quality. Therefore, more cautions must be put into the interpretation of the evaluation results.

**Table 4:** **Pearson correlation of the FM** (**template**-**free modeling**) **targets on Stage 1 of CASP10**

**Targets**	**MULTICOM-NOVEL**	**MULTICOM-CLUSTER**	**MULTICOM-CONSTRUCT**	**MULTICOM-REFINE**	**DAVIS-QA consensus**	**Pcons**	**ModFOLDclust2**
T0666	0.570	0.454	0.138	0.272	0.274	0.346	0.538
T0735	0.725	0.704	0.414	0.083	0.086	0.667	0.030
T0734	0.522	0.544	0.152	-0.099	-0.096	0.509	-0.014
T0737	0.878	0.878	0.221	0.118	0.124	0.565	0.421
T0740	0.558	0.512	0.710	0.732	0.726	0.684	0.770
T0741	-0.020	0.214	-0.659	-0.615	-0.611	0.475	-0.674
Average	0.539	0.551	0.163	0.082	0.084	0.541	0.179

**Table 5:** **Pearson correlation of all FM** (**template**-**free modeling**) **targets on Stage 2 of CASP10**

**Targets**	**MULTICOM-NOVEL**	**MULTICOM-CLUSTER**	**MULTICOM-CONSTRUCT**	**MULTICOM-REFINE**	**DAVIS-QA consensus**	**Pcons**	**ModFOLDclust2**
T0666	0.213	0.206	0.490	0.499	0.492	0.338	0.520
T0735	0.466	0.433	0.261	0.159	0.150	0.238	-0.070
T0734	0.459	0.44	-0.134	-0.342	-0.334	0.199	-0.363
T0737	0.787	0.806	0.200	0.155	0.147	0.583	0.525
T0740	0.490	0.451	0.487	0.412	0.411	0.434	0.478
T0741	-0.079	0.022	-0.444	-0.397	-0.397	0.125	-0.382
Average	0.389	0.393	0.143	0.081	0.078	0.320	0.118

Based on the per-target correlation between predicted and observed model quality scores of the official model quality assessment results [28], the MULTICOM-CONSTRUCT was ranked 5^th^ on Stage 2 models of CASP10 among all CASP10 model quality assessment methods. The performance of MULTICOM-CONSTRUCT was slightly better than the DAVIS-QAconsensus (the naïve consensus method that calculates the quality score of a model as the average structural similarity (GDT-TS score) between the model and other models in the pool) on Stage 2, which was ranked at 10^th^. The methods MULTICOM-REFINE, MULTICOM-NOVEL, and MULTICOM-CLUSTER were ranked at 11^th^, 28^th^, and 29^th^, respectively. However, it was not surprising that the single-model methods such as MULTICOM-NOVEL and MULTICOM-CLUSTER were ranked lower than most clustering-based methods because the latter tended to work better on most CASP template-based targets with good-quality predicted models. But, among all single-model methods, MULTICOM-NOVEL and MULTICOM-CLUSTER were ranked at 3^th^ and 4^th^.

### Results of local quality

Table 6 shows the performance of local quality assessment of our four local quality assessment servers, DAVIS-QAconsensus, Pcons, and ModFOLDclust2 on both Stage 1 and Stage 2. Among our four servers, the multi-model methods performed better than single-model methods on average for all the targets. We used the pairwise Wilcoxon signed ranked sum test to assess the significance of the difference between our four servers and the three external methods (Table 7). Generally speaking, the difference between multi-model local quality methods (MULTICOM-REFINE, DAVIS-QAconsensus, Pcons, and ModFOLDclust2) and single-model local quality methods (MULTICOM-NOVEL, MULTICOM-CLUSTER, MULTICOM-CONSTRUCT) on both stages is significant. The difference between MULTICOM-REFINE and Pcons is not significant on both stages according to a 0.01 threshold.

**Table 6:** **Evaluation result of local quality score of four MULTICOM servers**, **DAVIS**-**QAconsensus**, **Pcons**, **and ModFOLDclust2 on Stage 1 and Stage 2 of CASP10**

**Servers**	**Ave. corr. on Stage 1**	**Ave. corr. on Stage 2**
MULTICOM-REFINE	0.6102	0.6251
MULTICOM-CLUSTER	0.2604	0.2956
MULTICOM-NOVEL	0.2882	0.3289
MULTICOM-CONSTRUCT	0.2889	0.3095
DAVIS-QAconsensus	0.5841	0.6633
Pcons	0.5793	0.6226
ModFOLDclust2	0.5997	0.6526

**Table 7:** **The P**-**value of pairwise Wilcoxon signed ranked sum tests for the difference of correlation scores for local model quality prediction methods** (**MULTICOM servers**, **DAVIS**-**QAconsensus**, **Pcons**, **and ModFOLDclust2**)

**MULTICOM servers, DAVIS-QAconsensus, Pcons, and ModFOLDclust2 and on Stage 1 or Stage 2**	**P-value**
MULTICOM-REFINE and MULTICOM-CLUSTER on Stage 1	2.220e-16
MULTICOM-REFINE and MULTICOM-NOVEL on Stage 1	6.661e-16
MULTICOM-REFINE and MULTICOM-CONSTRUCT on Stage 1	6.661e-16
MULTICOM-CLUSTER and MULTICOM-NOVEL on Stage 1	0.0009948
MULTICOM-CLUSTER and MULTICOM-CONSTRUCT on Stage 1	0.0008437
MULTICOM-NOVEL and MULTICOM-CONSTRUCT on Stage 1	0.1781
MULTICOM-REFINE and Pcons on Stage 1	0.01575
MULTICOM-REFINE and ModFOLDclust2 on Stage 1	0.2678
MULTICOM-REFINE and DAVIS-QAconsensus on Stage 1	0.00699
MULTICOM-CLUSTER and Pcons on Stage 1	2.2e-16
MULTICOM-CLUSTER and ModFOLDclust2 on Stage 1	2.553e-16
MULTICOM-CLUSTER and DAVIS-QAconsensus on Stage 1	2.442e-15
MULTICOM-NOVEL and Pcons on Stage 1	2.2e-16
MULTICOM-NOVEL and ModFOLDclust2 on Stage 1	3.046e-16
MULTICOM-NOVEL and DAVIS-QAconsensus on Stage 1	4.885e-15
MULTICOM-CONSTRUCT and Pcons on Stage 1	2.2e-16
MULTICOM-CONSTRUCT and ModFOLDclust2 on Stage 1	3.137e-16
MULTICOM-CONSTRUCT and DAVIS-QAconsensus on Stage 1	4.78e-15
MULTICOM-REFINE and MULTICOM-CLUSTER on Stage 2	2.269e-16
MULTICOM-REFINE and MULTICOM-NOVEL on Stage 2	6.661e-16
MULTICOM-REFINE and MULTICOM-CONSTRUCT on Stage 2	3.137e-16
MULTICOM-CLUSTER and MULTICOM-NOVEL on Stage 2	0.00327
MULTICOM-CLUSTER and MULTICOM-CONSTRUCT on Stage 2	0.5493
MULTICOM-NOVEL and MULTICOM-CONSTRUCT on Stage 2	1.029e-14
MULTICOM-REFINE and Pcons on Stage 2	0.2498
MULTICOM-REFINE and ModFOLDclust2 on Stage 2	0.0005575
MULTICOM-REFINE and DAVIS-QAconsensus on Stage 2	2.443e-06
MULTICOM-CLUSTER and Pcons on Stage 2	2.220e-16
MULTICOM-CLUSTER and ModFOLDclust2 on Stage 2	2.220e-16
MULTICOM-CLUSTER and DAVIS-QAconsensus on Stage 2	2.220e-16
MULTICOM-NOVEL and Pcons on Stage 2	4.441e-16
MULTICOM-NOVEL and ModFOLDclust2 on Stage 2	2.220e-16
MULTICOM-NOVEL and DAVIS-QAconsensus on Stage 2	2.220e-16
MULTICOM-CONSTRUCT and Pcons on Stage 2	4.089e-16
MULTICOM-CONSTRUCT and ModFOLDclust2 on Stage 2	2.2e-16
MULTICOM-CONSTRUCT and DAVIS-QAconsensus on Stage 2	2.2e-16

However, the single-model local quality prediction methods (MULTICOM-NOVEL, MULTICOM-CLUSTER, MULTICOM-CONSTRUCT) and the multi-model local quality prediction method (MULTICOM-REFINE) performed not very differently on FM targets as shown in Tables 8 and 9. This is not surprising because multi-model methods cannot select real good models as reference methods for evaluating the local quality of residues.

**Table 8:** **Local quality score of four MULTICOM servers**, **DAVIS**-**QAconsensus**, **Pcons**, **and ModFOLDclust2 for all FM** (**template**-**free modeling**) **targets on Stage 1 of CASP10**

**Targets**	**MULTICOM-NOVEL**	**MULTICOM-CLUSTER**	**MULTICOM-CONSTRUCT**	**MULTICOM-REFINE**	**DAVIS-QA consensus**	**Pcons**	**ModFOLDclust2**
T0666	0.261	0.216	0.262	0.261	0.195	0.303	0.164
T0735	0.118	0.083	0.122	0.366	0.190	0.214	0.224
T0734	0.025	0.105	0.025	0.402	0.302	0.166	0.232
T0737	0.554	0.664	0.551	0	0.186	0.704	0.122
T0740	0.242	0.196	0.243	0.442	0.368	0.377	0.407
T0741	0.078	-0.035	0.084	0.227	0.108	-0.072	0.136
Average	0.213	0.205	0.215	0.283	0.225	0.282	0.214

**Table 9:** **Local quality score of four MULTICOM servers**, **DAVIS**-**QAconsensus**, **Pcons**, **and ModFOLDclust2 for all FM** (**template**-**free modeling**) **targets on Stage 2 of CASP10**

**Servers**	**MULTICOM-NOVEL**	**MULTICOM-CLUSTER**	**MULTICOM-CONSTRUCT**	**MULTICOM-REFINE**	**DAVIS-QA consensus**	**Pcons**	**ModFOLDclust2**
T0666	0.244	0.226	0.227	0.310	0.322	0.282	0.337
T0735	0.125	0.122	0.127	0.288	0.290	0.150	0.351
T0734	0.129	0.151	0.122	0.172	0.330	0.255	0.305
T0737	0.426	0.578	0.419	0	0.202	0.583	0
T0740	0.268	0.197	0.257	0.270	0.422	0.377	0.425
T0741	0.105	-0.011	0.109	0.165	0.129	0.009	0.119
Average	0.216	0.211	0.210	0.200	0.283	0.276	0.256

According to the CASP official evaluation [28], MULTICOM-REFINE performs best among all of our four servers for the local quality assessment on both Stage 1 and Stage 2 models of CASP10. Compared with DAVIS-QAconsensus, Pcons, and ModFOLDclust2, the multi-model local quality prediction method MULTICON-REFINE performed best on Stage 1, achieved the similar performance with Pcons on Stage 2, but performed worse than DAVIS-QAconsensus and ModFOLDclust2 on Stage 2.

## Conclusion

In this work, we rigorously benchmarked our multi-model and single-model quality assessment methods blindly tested in the Tenth Critical Assessment of Techniques for Protein Structure Prediction (CASP10). In general, the performance of our multi-model quality prediction methods (e.g., MULTICOM-REFINE) was comparable to the state-of-the-art multi-model quality assessment methods in the literature. The multi-model quality prediction methods performed better than the single-model quality prediction methods (e.g., MULTICOM-NOVEL, MULTICOM-CLUSTER), whereas the latter, despite in its early stage of development, tended to work better in assessing a small number of models of wide-range quality usually associated with a hard target. Our experiment demonstrated that the prediction accuracy of multi-model quality assessment methods is largely influenced by the proportion of good models in the pool or the average quality of the largest model cluster in the pool. The multi-model quality assessment methods performed better than single-model methods on easy modeling targets whose model pool contains a large portion of good models. However, they tend to fail on the models for hard targets when the majority of models are of low-quality and particularly when some low-quality models are similar to each other severely dominating the calculation of pairwise model similarity. The problem can be somewhat remedied by using single-model quality prediction scores as weights in calculating the average similarity scores between models. However, to completely address the problem, more accurate single-model quality prediction methods that can assess the quality of a single model need to be developed. On one hand, more informative features such as sequence conservation information, evolutionary coupling information, torsion angle information, and statistical contact potentials may be used to improve the discriminative power of single-model methods; on the other hand, new powerful machine learning and data mining methods such as deep learning, random forests and outlier detection methods may be developed to use existing quality features more effectively. Despite it may take years for single-model methods to mature, we believe that improved single-model quality prediction methods will play a more and more important role in protein structure prediction.

## Methods

### Protein model quality prediction methods

The methods used by the four automated protein model quality assessment servers are briefly described as follows.

**MULTICOM-REFINE** is a multi-model quality assessment method using a pairwise model comparison approach (APOLLO) [29] to generate global quality scores. The 19 top models based on the global quality scores and the top 1 model selected by SPICKER [32] formed a top model set for local quality prediction. After superimposing a model with each model in the top model set, it calculated the average absolute Euclidean distance between the position of each residue in the model and that of its counterpart in each model in the top model set. The average distance was used as the local quality of each residue.

**MULTICOM-CLUSTER** is a single-model, support vector machine (SVM)-based method initially implemented in [24]. The input features to the SVM include a window of amino acids encoded by a 20-digit vector of 0 and 1 centered on a target residue, the difference between secondary structure and solvent accessibility predicted by SCRATCH [33] from the protein sequence and that of a model parsed by DSSP [34], and predicted contact probabilities between the target residue and its spatially neighboring residues. The SVM was trained to predict the local quality score (i.e. the Euclidean distance between its position in the model and that in the native structure) of each residue. The predicted local quality scores of all the residues was converted into the global quality score of the model according to the formula [35] as follows:

$$\mathrm{Global}\;\mathrm{quality}\;\mathrm{score}=\frac{1}{L}\sum_{i=1}^{t}\left(\frac{1}{1+{\left(\frac{S_{i}}{T}\right)}^{2}}\right)$$

In the formula, *L* is the total number of residues, *S*_*i*_ is the local quality score of residue i, and T is a distance threshold set to set to 5 Angstrom. Residues that did not have a predicted local quality score were skipped in averaging.

**MULTICOM-NOVEL** is the same as MULTICOM-CLUSTER except that amino acid sequence features were replaced with the sequence profile features. The multiple sequence alignment of a target protein used to construct profiles was generated by PSI-BLAST [36].

**MULTICOM-CONSTRUCT** uses a new, weighted pairwise model evaluation approach to predict global quality. It uses ModelEvaluator [37] – an ab initio single-model global quality prediction method – to predict a score for each model, and uses TM-score [35] to get the GDT-TS score for each pair of models. The predicted global quality score of a model *i* is the weighted average GDT-TS score between the model and other models, calculated according to the formula: $S_{i}=\sum_{j=1}^{N}\left(X_{i,j}*\frac{W_{J}}{\sum_{j=1}^{N}W_{j}}\right)$. In this formula, *S*_*i*_ is the predicted global quality score for model *i*, *N* is the total number of models, *X*_*i,j*_ is the GDT-TS score between model *i* and model *j*, *W*_*j*_ is the score for model *j* predicted by ModelEvaluator, which is used to weight the contribution of X_i,j_ to *S*_*i*_. In case that no score was predicted for a model by ModelEvaluator, the weight of the model is set to the average of all the scores predicted by ModelEvaluator. The local quality prediction of MULTICOM-CONSTRUCT is the same as MULTICOM-NOVEL except that additional SOV (segment overlap measure of secondary structure) score features were used by the SVM to generate the local quality score.

### Evaluation methods

CASP10 used two-stage experiments to benchmark for model quality assessment. Stage 1 had 20 models with different qualities for each target, and Stage 2 had 150 top models for each target selected from all the models by a naïve pairwise model quality assessment method. We downloaded the native structures of 98 CASP10 targets, their structural models, and the quality predictions of these models made by our four servers during the CASP10 experiment running from May to August, 2012 from the CASP website (http://predictioncenter.org/casp10/index.cgi).

We used TM-score [35] to calculate the real GDT-TS scores between the native structures and the predicted model as their real global quality scores. The predicted global quality scores of our four servers were used to compare with the real global quality scores. In order to calculate real local quality scores of residues in a model, we first used TM-score to superimpose the native structure and the model, and then calculate the Euclidean distance between each residue’s coordinates in the superimposed native structure and the model as the real local quality score of the residue. The real local and global quality scores of a model were compared with that predicted by the model quality assessment methods to evaluate their prediction accuracy.

We evaluated the global quality of our predictions from five aspects: the average of per-target Pearson correlations, the overall Pearson’s correlation, average GDT-TS loss, the average Spearman’s correlation, and the average Kendall tau correlation. The average of per-target Pearson’s correlations is calculated as the average of all 98 targets’ Pearson correlations between predicted and real global quality scores of their models. The overall Pearson’s correlation is the correlation between predicted and real global quality scores of all the models of all the targets pooled together. The average GDT-TS loss is the average difference between the GDT-TS scores of the real top 1 model and the predicted top 1 model of all targets, which measures how well a method ranks good models at the top. The Spearman’s correlation is the Pearson’s correlation of the ranked global quality scores. In order to calculate the Spearman’s rank correlation, we first convert the global quality scores into the ranks. The identical values (rank ties or duplicate values) are assigned a rank equal to the average of their positions in the rank list. And then we calculate the Pearson’s correlation between the predicted ranks and true ranks of the models. The Kendall tau correlation is the probability of concordance minus the probability of discordance. For two vectors x and y with global quality scores of n models of a target, the number of total possible model pairs for x or y is $N=\frac{n*\left(n-1\right)}{2}$. The number of concordance is the number of pairs and (*X*_*j*_,*Y*_*j*_) when (*x*_*i*_ - *x*_*j*_) * (*y*_*i*_ - *y*_*i*_) > 0, and the number of discordance is the number of pairs *X*_*i*_,*Y*_*i*_ and (*X*_*j*_,*Y*_*j*_) when (*x*_*i*_ - *x*_*j*_) * (*y*_*i*_ - *y*_*i*_) < 0. The Kendall tau correlation is equal to the number of concordance minus the number of discordance divided by N. (http://en.wikipedia.org/wiki/Kendall_tau_rank_correlation_coefficient).

The accuracy of local quality predictions was calculated as the average of the Pearson’s correlations between predicted local quality scores and real local quality scores of all the models of all the targets. For each model, we used TM-score to superimpose it with the native structure, and then calculated the Euclidean distance between Ca atom’s coordinates of each residue in a superimposed model and the native structure as the real local quality score of each residue. The Pearson’s correlation between the real quality scores and the predicted ones of all the residues in each model was calculated. The average of the Pearson’s correlations of all the models for all 98 targets was used to evaluate the performance of the local quality prediction methods.

## Competing interests

The authors declare that they have no competing interests.

## Authors’ contributions

JC conceived and designed the method and the system. RC, ZW implemented the method, built the system, carried out the CASP experiments. RC, ZW, JC evaluated and analyzed data. RC, JC wrote the manuscript. All the authors approved the manuscript.
